# Risk Factor Profile for Cardiovascular Diseases Among Farmhouse Residents in Vijayapura District, Karnataka: A Cross-Sectional Study

**DOI:** 10.7759/cureus.66748

**Published:** 2024-08-13

**Authors:** Sandeep G Yankanchi, Rekha Udgiri, Praveen Ganganahalli, Santosh D Patil

**Affiliations:** 1 Community Medicine, Shri B. M. Patil Medical College Hospital and Research Centre, BLDE (Deemed to be University), Vijayapura, IND

**Keywords:** agriculture, cardiovascular disease, farmhouse, risk factor, vijayapura

## Abstract

Background

Cardiovascular illnesses are widely regarded as the leading cause of mortality worldwide, with 17.9 million deaths every year. Rheumatic heart disease, coronary heart disease, and cerebrovascular illness are among the conditions relating to the heart and blood vessels together, known as cardiovascular diseases. The main behavioural risk factors for heart disease and stroke include poor diet, inactivity, tobacco use, and excessive alcohol consumption. In contrast, one of the significant environmental risk factors is air pollution. People may experience high blood pressure and high blood sugar due to obesity and overweight as risk factors.

Objectives

This study aims to find the risk factor profile for cardiovascular diseases among the farmhouse residents of the Vijayapura district, India.

Materials and methods

A cross-sectional descriptive study was conducted with the farmhouse inhabitants of the Vijayapura district. Using a prestructured proforma that included details on the sociodemographic profile and risk factors of the farmhouse dwellers, 450 sample participants were questioned. Five families within a chosen primary sampling unit (PSU) were chosen randomly from a pool of PSUs that were picked using a probability proportional to size sampling technique. All characteristics were summarized descriptively.

Results

The study included 450 participants, 50.9% males and 49.1% females. The age range of 21.4% of the male participants was between 41 and 50, whereas 21.7% of the female participants belonged to the 41 to 50 age group. The study showed a proportion of them had the habit of alcohol consumption (5.8%), chewing tobacco (2%), and smoking (1.6%). Compared to female participants (1.3% vs. 1.3%), the majority of male individuals (4.8% vs. 7%) had hypertension and diabetes mellitus, respectively.

Conclusion

The findings of the current research indicated that the majority of farm inhabitants in the rural Vijayapura district were illiterate, belonged to a lower socioeconomic class, and had intermediate and behavioural risk factors for cardiovascular illnesses.

## Introduction

Cardiovascular illnesses are widely regarded as the leading cause of mortality worldwide, with 17.9 million deaths every year. Conditions related to the heart and blood vessels are cardiovascular diseases, rheumatic heart disease, coronary heart disease, and cerebrovascular disease. Tobacco use, alcohol consumption, physical inactivity, and a poor diet are the main behavioural risk factors for heart disease and stroke. One significant risk factor for the environment is air pollution. Individuals may have behavioural risk factors such as elevated blood pressure, high blood sugar, obesity, and being overweight. In primary healthcare settings, these "intermediate risk factors" can be assessed and show an elevated risk of heart attack, stroke, heart failure, and other consequences [1].

A "farmhouse" is a dwelling built on a piece of land used for farming, serving as either the residence of the farmer or a storage facility for farming equipment and cattle. The farmer can utilize the house for personal use only; no other individual or organization may use it for their benefit [2].

The health services might not reach the farmhouse workers because they are widely scattered in rural locations. By identifying those at the highest risk of cardiovascular illnesses and ensuring they receive the appropriate care, premature deaths can be prevented. More information on cardiovascular disease risk factors needs to be provided to those who live in farmhouses. Studies based on communities can only capture the whole picture of risk variables within those communities. The current study investigated the risk factors for cardiovascular disease among the farmhouse dwellers of the Vijayapura district.

## Materials and methods

Study design and setting

This observational cross-sectional study was conducted in the Vijayapura district in the northern part of Karnataka state [3]. The study was conducted among household members staying in farmhouses for a period of one year (June 2017 to May 2018) using the interview technique with a pretested, semi-structured questionnaire. The questionnaire was prepared and validated by subject experts and pilot-tested in the rural field practice area of the Community Medicine department.

Inclusion criteria

Participants who have lived in farmhouses for more than six months or are permanent residents of the farmhouse and belong to the age group of more than 19 years were included in the study.

Exclusion criteria

The study excluded individuals who were residents of farmhouses but migrated to other places for employment purposes, as well as those who had not given consent for participation and had a documented history of cardiovascular diseases and diabetes mellitus.

Study tool

We gathered data on risk factors such as age, education, alcohol intake, and tobacco use (smoking or chewing) using a standardized questionnaire. Blood glucose, blood pressure, and haemoglobin estimations were done using an Accu-Chek glucometer (Roche Diabetes Care, Inc., Indianapolis, Indiana) [4], a Mission Plus Hb meter (ACON Laboratories, San Diego, California) [5], and a digital blood pressure monitor [6].

The Institutional Ethics Committee approval (BLDE(DU)/IEC/61/2016) and informed consent from the participants were obtained before we began the data collection.

Study method

Household selection was done at different stages within each taluka (total of five taluka), using villages as a primary sampling unit (PSU) [7]. The villages with less than five farmhouse families were excluded from the list. There are two stages of household selection. Five households within each PSU were chosen randomly after PSUs were chosen using probability proportional to size (PPS) sampling. The government primary health centre provided a list of the households residing on the farm, and chits with the name of the family head were made. Five chits from each village were chosen randomly for the study. Prestructured questionnaires were used to randomly ask four participants in each household, aged over 19 years, regarding the risk factor profile for cardiovascular diseases among the farmhouse residents.

Sample size calculation

The sample size was determined using the formula n= z2pq/d2. The calculation was predicated on a 50% prevalence assumption because insufficient data were available regarding the risk factor profile for cardiovascular illnesses among farm dwellers in the research area. With a 95% confidence level and 5% accuracy assumption, 384 farm residents represented the total sample size, but the total enrollment was 450.

Statistical analysis

The chi-square (χ2) test was used to determine the significance of differences between groups for categorical data. Data were analyzed using Microsoft Excel (Microsoft Corporation, Redmond, Washington) and IBM SPSS Statistics for Windows, Version 23 (Released 2015; IBM Corp., Armonk, New York) with a 95% confidence interval, and a p-value of less than 0.05 was considered significant.

## Results

**Figure 1:**
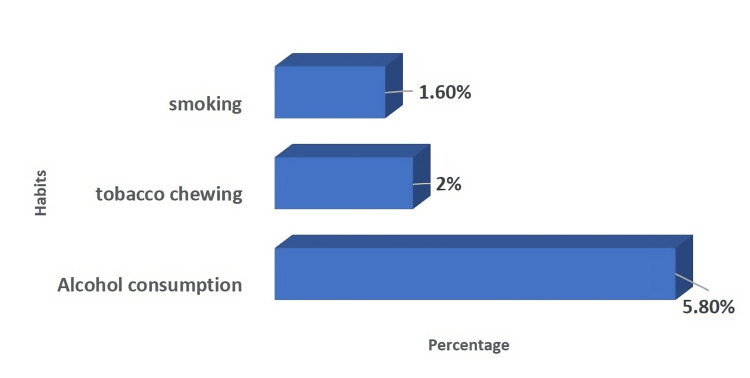
Distributions of participants according to habits (N=450)

A total of 450 participants were included in the study; 50.9% were men, and 49.1% were women. The age range of 21.4% of the male participants was between 41 and 50 years old, whereas 21.7% of the female participants belonged to the 41 to 50-year age group. The study showed that the majority of them had the habit of alcohol consumption (5.8%), followed by chewing tobacco (2%) and smoking (1.6%), as depicted in Figure 1.

More than 70% of male and female participants were within the normal range of 18.5 to 24.99 body mass index (BMI) range. Compared to female participants (24, 11.6%), the majority of male participants (59, 24.3%) were underweight (<18.5). Out of all the participants, more women, 26 (12.6%) vs. 2 (1.1%), were overweight or obese than males, 10 and 1 (4.3%, 0.5%), respectively (Table 1).

**Table 1:** Distribution of participants according to body mass index (BMI) (N=450) χ2-value = 17.885, at a 5% level of significance (p<0.05)

BMI Status	Males		Females	
	N	%	N	%
Underweight	59	24.3	24	11.6
Normal	173	71.0	155	74.7
Overweight	10	4.3	26	12.6
Obese	1	0.5	2	1.1
Total	243	100	207	100

Following a random blood sugar test, 373 (82.8%) had normal blood sugar levels, whereas 14 (3.2%) already had diabetes mellitus (males more than females). During the investigation, 63 (14%) were found in the prediabetic range (females more than males). The proportion distribution was found to be statistically significant (Table 2).

**Table 2:** Distribution of participants based on blood sugar level (N=450) *Significant χ2-value = 6.503, at a 5% level of significance (p<0.05)

Diabetes Mellitus	Male		Female		Total		p-value
	N	%	N	%	N	%	
Normal	205	84.4	168	81	373	82.8	0.0387*
Prediabetic	26	10.8	37	17.7	63	14	
Diabetic	12	4.8	2	1.3	14	3.2	
Total	243	100	207	100	450	100	

Following a blood pressure examination, it was seen that 20 (4.6%) of participants had Grade 1 hypertension (males more than females), whereas 40.8% (females more than males) were found in the pre-hypertension range. The distribution of the proportions was found to be statistically significant (p<0.05) (Table 3).

**Table 3:** Distribution of participants based on blood pressure level (N=450) *Significant χ2-value = 13.074, at a 5% level of significance (p<0.05)

Blood Pressure	Male		Female		Total		p-value
	N	%	N	%	N	%	
Normal	144	59.1	102	49.3	246	54.6	0.0014*
Prehypertension	82	33.9	102	49.3	184	40.8	
Grade I hypertension	17	7.0	3	1.4	20	4.6	
Total	243	100.0	207	100.0	450	100.0	

When haemoglobin estimation was done, the majority of the participants, 284 (63.1%), had normal haemoglobin levels (>11 g%), but surprisingly, the majority of male participants, 117 (48.3%), had mild anaemia (9-11 g%) compared to females. However, none of them had severe anaemia (<7 g%). The proportion of distribution was found to be statistically significant (p<0.05). According to Table 4, the most common risk factor among farmhouse residents was the prehypertension stage (41%), followed by their age of more than 40 years (23%).

**Table 4:** Distribution of participants according to the risk factors (N=450)

Risk Factors	Present		Absent	
	N	%	N	%
Age (41–50 years)	102	23	348	77
BMI>25	39	9	411	91
Alcohol consumption	26	5.80	424	94.2
Tobacco chewing	9	2	441	98
Smoking	7	1.6	443	98.4
Prediabetics	63	14	387	86
Prehypertension	184	41	266	59

## Discussion

Agricultural workers are exposed to numerous risk factors. However, this fact is sometimes overlooked due to the pervasive belief that occupational health issues are primarily related to industry and developed nations. As a result, there are extremely few study articles about farmhouse residents.

According to the results of the current study, 1.6% of the farmhouse residents were smokers, 2% chewed tobacco, and 5.8% of them were alcoholics. In a rural area of Vijayapura, the majority of participants were smokers (18.8%) and alcoholics (23.5%), according to the District Level Household and Facility Survey (DLHS-4) study [8]. According to Karmakar et al., smoking (31.2%) was the most prevalent addiction in rural areas. By contrast, 12.7% of them suffered from alcohol addiction, and 13.8% of them smoked tobacco (chewable varieties such as gutkha and khaini) [9]. Compared to earlier investigations, the current study revealed reduced levels of addictive behaviour, which may have been caused by less access to alcohol and lower socioeconomic status.

According to the results of the current study, compared to female participants (11.6%), the majority of male participants, aged 41 to 50, were underweight (24.3%). Women were more likely than men to be overweight (12.6%) and obese (1.1%), with rates of 4.3% and 0.5%, respectively. According to the National Family Health Survey (NFHS-4) (Karnataka) study, the majority of female participants (24.3%) were underweight compared to male participants (17.6%). Of the participants, 35% of children under the age of five were underweight, and the majority of men (17.1%) were overweight and obese compared to girls (16.6%) [10]. This disparity between the current study and another investigation could result from participant changes in lifestyle, ignorance, or a lack of awareness regarding nutritional status in farming settings. According to the study conducted by Hameed et al., females had significantly greater rates of arthritis, anaemia, and obesity than male participants [11].

A total of 3.2% of the participants in the current study had diabetes mellitus. Compared to female participants (1.3%), the majority of male participants (4.8%) had diabetes mellitus, and 4.4% had Grade 1 hypertension. Compared to female participants (1.3%), the majority of male individuals, 13 (7%), had Grade I hypertension. Agarwal et al. estimated that 7% of rural Agra residents have diabetes mellitus [12]. According to Gupta et al.'s findings, 5.99% of those surveyed in rural Tamil Nadu had diabetes [13]. Similar results were found by Satheesh et al. in rural Karnataka's coastal areas, where the percentage of people with hypertension was higher and the overall prevalence of hypertension was 18% [14]. According to Raja et al., 26.2% of adults in Tamil Nadu's rural Kanchipuram region had hypertension. Men were at a higher risk than women (OR=1.390) [15]. The discrepancy between the results of the current study and those of previous studies may be explained by the farmers' hard work, higher levels of physical exercise, and their stress-free work environment.

Strength of the study

There is a severe lack of research articles regarding the health status of the residents living in farmhouses. Therefore, our study contributes to filling the existing research gap in this field.

Limitation of the study

The present research was conducted in a secluded area within the northern part of Karnataka state. Therefore, the study findings cannot be generalizable to all farmhouse residents in the remaining parts of the states and the country.

## Conclusions

Agriculture is a longstanding and crucial sector in our country, with 80% of the population residing in rural areas where agriculture is the primary occupation. Farmhouse labourers are a unique population widely distributed in rural locations, making it difficult for them to get health treatments. The scientific literature contains very little realistic data on the risk factors for cardiovascular disease among farmhouse residents. The current study of farm dwellers in the rural area of Vijayapura district found that the majority were illiterate, from a lower socioeconomic level, and had behavioural risk factors such as alcohol intake, tobacco chewing, and smoking. The majority of the participants had intermediate risk factors, such as prediabetic and prehypertension stages and obesity. Limited access to primary healthcare services may contribute to increased risk factors for cardiovascular diseases among farmhouse residents.

## Appendices

**Table 5:** Questionnaire

Sociodemographic Characteristics	Name of the Household
	Address
	Religion
	Socioeconomic status
	Family composition
	Type of family
General physical examination	
Personal habits	Smoking
	Alcohol
	Tobacco chewing
	Others
Vital signs	Pulse rate/min
	Blood pressure (mmHg)
Anthropometry	Height (cm)
	Weight (kg)
	BMI weight (kg)/height (m^2^)
Investigations	Random blood sugar (mg/dL)
	Haemoglobin in g %
Systemic examination	
